# Why did only one genus of insects, *Halobates*, take to the high seas?

**DOI:** 10.1371/journal.pbio.3001570

**Published:** 2022-04-13

**Authors:** Lanna Cheng, Himanshu Mishra

**Affiliations:** 1 Scripps Institution of Oceanography, University of California San Diego, La Jolla, California, United States of America; 2 Environmental Science and Engineering Program, Biological and Environmental Science and Engineering Division, Water Desalination and Reuse Center, King Abdullah University of Science and Technology, Thuwal, Saudi Arabia

## Abstract

Oceans cover more than 70% of the Earth’s surface and house a dizzying array of organisms. Mammals, birds, and all manner of fish can be commonly sighted at sea, but insects, the world’s most common animals, seem to be completely absent. Appearances can deceive, however, as 5 species of the ocean skater *Halobates* live exclusively at the ocean surface. Discovered 200 years ago, these peppercorn-sized insects remain rather mysterious. How do they cope with life at the ocean surface, and why are they the only genus of insects to have taken to the high seas?

## Abstract 2

Oceans cover over 70% of the earth’s surface and house a dizzying array of organisms, including five species of the peppercorn-sized ocean-skater Halobates, which live exclusively at the ocean surface. How do they cope with life at the ocean surface and why are they the only genus of insects able to conquer the high seas?

## Introduction

We can’t avoid insects during our daily lives. They are the most numerous animals on Earth and occur everywhere. We see them all the time and find them in every conceivable nook and cranny, yet we have been led to believe that there are no insects in the open ocean. This belief is incorrect, as there are actually 5 insect species in the genus *Halobates* (Heteroptera: Gerridae) that live exclusively on the open ocean surface hundreds of kilometers away from land [1]. They are related to the water skaters commonly found in freshwater ponds, lakes, and streams but are almost unknown because the adults are around 4 mm long (about the size of a peppercorn) and are too small to be seen from ships. Even Charles Darwin did not see them during his voyage on the HMS Beagle (1831 to 1836) [2].

Ocean insects were first collected during the Russian circumnavigation expedition (1815 to 1818) on the warship Rurik to discover and explore the Northwest Passage. Johann Friedrich Eschscholtz, the ship’s doctor and a naturalist, described the genus *Halobates* with 3 new species (*Halobates micans*, *Halobates sericeus*, and *Halobates flaviventris*), together with 82 other new insect species in an entomological journal in 1822 [3]. Following their discovery during the Rurik expedition, *Halobates* specimens were collected in many subsequent oceanographic expeditions. The first review, which described 11 species of *Halobates*, was based on collections made during the Challenger Expedition (1872 to 1876) and was published in 1883 [4]. However, the literature on *Halobates* remained scattered and replete with errors and misidentifications until Jon Herring produced the first detailed and authoritative review on *Halobates* in 1961, with an annotated list of all previously published literature [5]. Based on the distributions of all the species described, Herring divided *Halobates* into 2 distinct groups, open ocean and coastal, and laid the foundation for future research.

The 5 open ocean species of *Halobates* constitute a mere 0.0001% of all insect species known, and yet they occupy almost half of the surface of the 3 major oceans. Why are there not more ocean-dwelling insects, and how have these ocean skaters managed to carve out a niche for themselves on the open oceans? Here is the story of the curious *Halobates*.

## Why are there so few marine insects?

**Fig 1:**
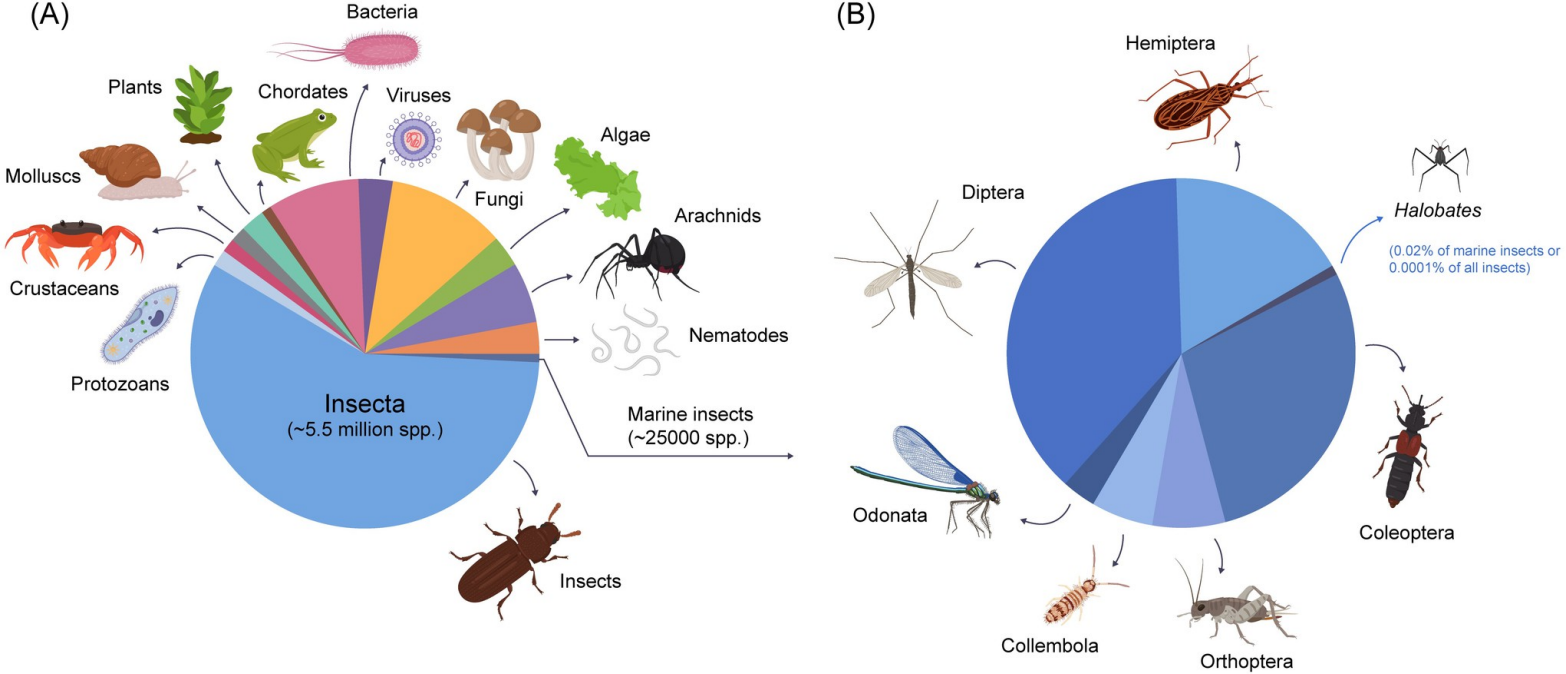
Pie charts showing proportions. **(A)** Estimated proportions of living organisms on Earth of which >70% are insects (approximately 5.5 million species), but only a tiny fraction (<0.05%) are considered marine, occurring in habitats ranging from brackish salt marshes, mangroves, and intertidal reef flats out to the open ocean. **(B)** Proportion of 6 major marine insect orders, of which approximately 20% are Hemiptera to which *Halobates* (approximately 50 species known; only 5 are oceanic) belongs. *This illustration was created by Ella Maru Studio*.

Insects emerged after the Cambrian explosion some 480 to 500 million years ago (Mya) when the seas were warm and shallow. Among the ultimate achievements of their evolutionary history are the development of wings, which enable them to disperse over long distances, and complete metamorphoses (with larval and pupal stages occupying different habitats from adults), which allow different life stages to occupy vastly different habitats. As a result, insects became the most successful organisms on land. To return to life in water, they needed to overcome various physiological constraints (such as underwater respiration and osmotic regulation) and physical constraints (such as buoyancy and surface tension), besides the reduction or disposal of wings, which do not function underwater. Insects evidently had no problems in readapting, and around 3% of total known insects are aquatic. Although only a tiny fraction of aquatic insects is considered marine, representatives of more than half of the 35 or so insect orders spend some stage of their life history in a marine habitat [6]. Of these, we guesstimate that some 25,000 species may be found in various saline habitats, most belonging to the orders Coleoptera, Diptera, and Hemiptera (Fig 1).

Why so few insects are marine has been a question of much debate and speculation, but no clear answers have emerged to date. Although it has been hypothesized that insects may not be able to overcome the physiological and physical constraints necessary to live in the sea, this does not appear to be the case, as brine flies can tolerate hypersaline waters, and several water beetles and water skaters are found in torrential streams [7–10]. The most likely hypothesis is that by the time insects evolved, the seas had already been well populated by all major phyla of marine invertebrates, which evolved some 200 million years earlier, and it may be that “osmotic regulation and submarine respiration involve evolution of such different physiological adaptations that few insects have been successful in achieving both goals” [7].

## How did *Halobates* conquer the open ocean?

Of the known marine insects, all require land (for food, to mate, to lay eggs, or to complete their life cycle) except for the 5 ocean-dwelling species in the genus *Halobates* [1]. This genus is unique in being not only the sole insect genus to have conquered the open ocean but also the only animal able to thrive at the ocean surface [11]. Unlike other animals living in the upper layers of the sea surface [12], such as the Portuguese man o’ war (*Physalia*), the “by-the-wind sailor” (*Velella*), the blue sea slug (*Glaucus*), and the stalked barnacle (*Dosima*), which float passively with their movements controlled entirely by ocean currents and winds, 5 *Halobates* species move freely over the ocean surface [11]. Being completely wingless, they live entirely at the ocean’s skin supported by the surface tension of water.

**Fig 2:**
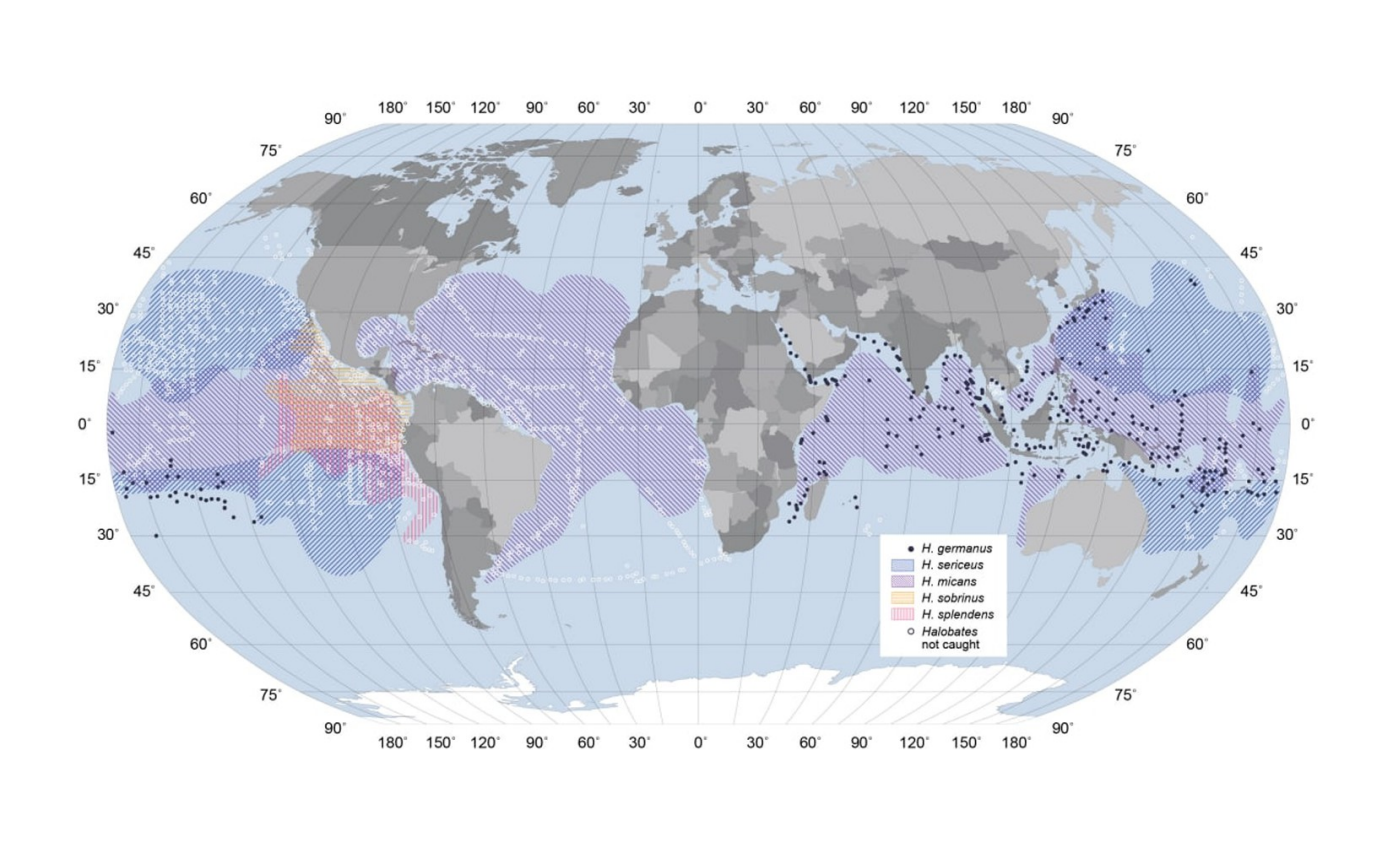
World Distribution of *Halobates*. The map shown here was compiled with data from >3,000 samples (LC Collections deposited at ZMUC, now known as Natural History Museum of Denmark) described in [19–21]. Ranges are shown for all species except *Halobates germanus*, which occurs widely in the Pacific as well the Indian Ocean and seems to prefer more coastal waters. Its distribution is presented as black dots where it has been caught. This difference in presentation is to aid interpretation of the data, as the ranges of the other 4 species overlap in many regions. White open circles represent known stations where no insects were caught. Note: very few collections were made in south Atlantic, Indian or eastern Pacific Oceans. We do not know if *Halobates* were actually absent from these areas. *Halobates micans* is cosmopolitan and widely distributed between latitudes 40N and 40S in the Atlantic but only extends to within 15S in the Indian Oceans. It’s range is restricted roughly between 15 N and 15 S in central Pacific Ocean where areas beyond its northern and southern boundaries are occupied by *Halobates sericeus* [20]. *Halobates sobrinus* and *Halobates splendens* (the rarest of the five species) are both confined to the eastern Pacific Ocean.

The 5 oceanic *Halobates* species (*H*. *micans*, *H*. *sericeus*, *Halobates germanus*, *Halobates sobrinus*, and *Halobates splendens*) can be found on almost half the ocean surface around the globe, making them one of the most widely distributed insects in the world. The distribution range of each species is distinct and well defined (Fig 2); however, which paleo-oceanographic factors were involved in determining how the 5 extant species got to where they are today and how their distinct distribution patterns are maintained are still not quite understood [13–15]. Ocean-dwelling *Halobates* tend to form large aggregates with patchy distributions. Although their ranges must be influenced by physical factors such as ocean circulation, prevailing winds and sea surface temperatures, they may also be able to skate with or against such forces [16]. It is usually quite impossible to spot *Halobates* from a ship’s deck because of the small size of the adults. However, on a calm day, it is just possible to spot them from the deck as tiny silvery balls rolling over the sea surface. The adults can skate at 1 ms^−1^ and jump to a height of 5 cm or more [10]. They also have very good eyesight and are able to see approaching nets towed from research vessels in daylight [17] and on full moon nights [18]. They can occasionally be found entangled in seaweeds washed ashore after strong tropical storms.

In addition to the 5 oceanic species, more than 40 coastal *Halobates* species are found along tropical island shores among mangroves or other coastal vegetation. Although open ocean and coastal *Halobates* share many characteristics [21], the most crucial adaptations for ocean skaters are their complete independence from land to complete their life history and their ability to cope with continuous exposure to sunshine and storms at sea.

## When did the ocean-dwelling lifestyle evolve?

Most likely, oceanic *Halobates* evolved from an estuarine or mangrove ancestor that got washed to sea and became adapted to survive in the open ocean. A fossil *Halobates ruffoi* discovered in the Pesciara di Bolca marine deposit in Verona, Italy dates back to about 45 Mya, indicating that coastal habitats had already been invaded by then. The first molecular phylogeny study on *Halobates*, published in 2008, was based on the mitochondrial gene COI and indicated that the move to an open ocean lifestyle probably occurred twice [22]. By contrast, a more recent study based on multiple genes indicated that the marine lifestyle evolved only once in the ancestral line of *Halobates*, but that open ocean life could have evolved 3 different times [23]. Clearly, more in-depth analyses are still needed to determine when and where the first open ocean *Halobates* originated and how the 5 extant oceanic species evolved.

## How is their life cycle adapted to ocean living?

*Halobates* goes through 5 nymphal stages before becoming adults. Analyses of samples collected at sea have shown that there is no distinct seasonality. The eggs, which are <1 mm long and shaped like miniature rice grains, are laid on any floating material the female can find, such as seeds, wood, seabird feathers, mollusk shells, and pieces of plastic [24]. The largest number of eggs recorded to date on a single substrate was on a plastic gallon milk jug, found off the coast of Costa Rica where *H*. *sobrinus* occurs, covered by an estimated 70,000 eggs laid 15 layers thick [25]; oviposition substrates are clearly hard to come by in the open ocean. The eggs take up to 10 days to hatch, and each nymphal stage may take 7 to 14 days, depending on water temperature. Thus, the development from egg to adult may take 2 months or more. How long adults can live in the open ocean is unknown [21].

Ocean skaters are fluid feeders and will feed on any small organism they can catch at the sea surface. Although their prey are marine and they may drink seawater, *H*. *sobrinus* can survive for up to 4 days on 0.5× and 1.5× salinity seawater, indicating that it can deal with osmoregulation [21]. We do not know what the newly hatched nymphs (measuring approximately 1 mm in length) feed on. They may obtain nutrients from seawater until they grow big enough to hunt. Given that large numbers of eggs are often found on a single floating substrate and can hatch at the same time in a patch, the young nymphs may survive by cannibalism. Food can be more difficult to come by in the open ocean than near the coast, where terrestrial insects are abundant prey, and in contrast to their coastal relatives, ocean skaters are able to store triglycerides as energy storage to tie them over short periods of starvation [26]. Although cannibalism is practiced among ocean skaters, and adults will feed readily on younger nymphs, we still do not know what their major prey items are in nature. As fluid feeders, it is unfortunately not possible to determine their prey by examining gut contents.

A little more is known about what predates on *Halobates*, as remains have been found in the guts of a few fish and marine turtles, but they are difficult for such predators to catch. Their commonest and most important predators are seabirds that feed by skimming over the ocean surface [27]. For example, remains of *H*. *sericeus* were found in 80% of 111 regurgitates from the blue-gray noddy, *Procelsterna cerulea*, with 336 insects in one sample, suggesting that they might be a major source of food for this small seabird during its breeding season [28].

In general, oceanic *Halobates* seem to have evolved a more extended life history strategy, compared with their freshwater relatives, which would allow them time to find mates and to reproduce even if they were widely dispersed from their hatching area by turbulence [16]. Yet, an extended life history strategy can only allow insects to colonize the open ocean if they can survive there.

## How are *Halobates* adapted to ocean life?

**Fig 3:**
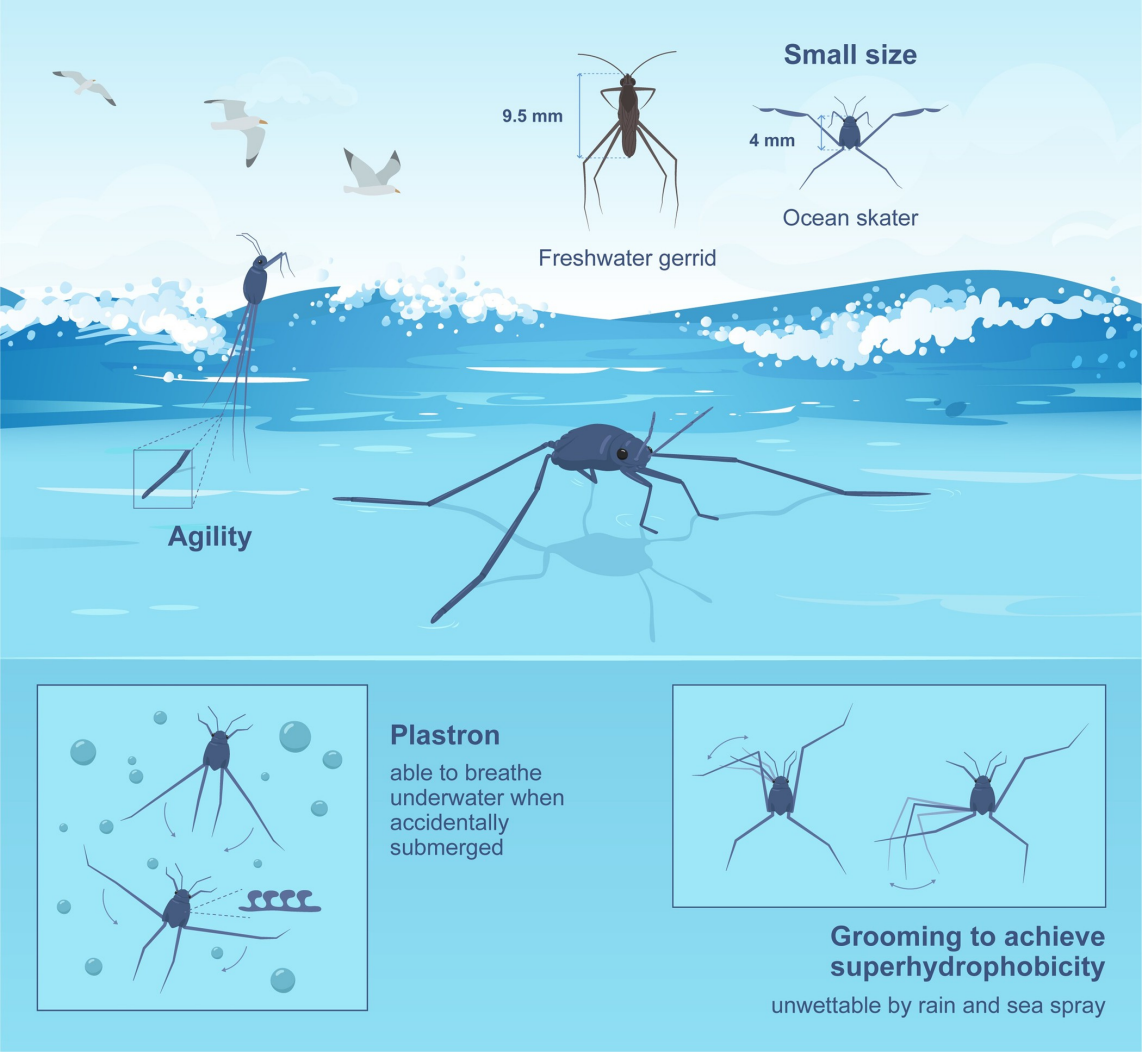
Key adaptations of ocean skaters (*Halobates*). Clockwise from the top right corner. *Halobates* have a small size and reduced body length/width ratio. They are oval and less than half the size of common freshwater skaters but with very similar leg lengths. This adaptation facilitates faster accelerations and higher speeds when moving. Frequent grooming to spread waxy secretion over their body hairs renders them superhydrophobic (or ultra-water repellent), which prevents wetting by sea-sprays and raindrops, allowing *Halobates* to fully exploit the water surface tension for jumping and skating. When accidentally submerged, the club-shaped hairs (microtrichia) covering their body entrap a layer of air (known as the plastron), allowing *Halobates* to breathe underwater and to resurface. They are also incredibly agile and are able to achieve impressive acceleration and height to evade dangers. *Illustration adapted from a figure created by Xavier Pita in Mahadik and colleagues [29]*.

Being the only insect able to live on the high seas, the survival of *Halobates* species requires the ability to evolve unique adaptations hitherto unknown in the insect world (Fig 3). Small size and an oval body seem to be the ultimate goals in the evolution of oceanic *Halobates*. Compared with the freshwater *Gerris* (subfamily Gerrinae), which measures about 9.5 mm in length and has a body length/width (*l*/*w*) ratio of approximately 5, *Halobates* (subfamily Halobatinae) measures about 4 mm in length with a *l/w* ratio of approximately 2 [29].

When viewed under a scanning electron microscope, the body of a *Halobates* is covered by densely packed microtrichia (approximately 1 μm high), small hairs that look like tiny mushrooms from above but are club-shaped from the side [30]. *Halobates* groom constantly when resting (as observed in other insects [31]) to spread waxy secretions over their bodies. Their wax-coated hairs prevent water from wetting them and also entrap air at the leg–water interface. This combination of nanoscale and microscale body hair covering and water-repellent wax coating endows *Halobates* with super-water repellence or superhydrophobicity [32].

When skating on water, these superhydrophobic ocean skaters are practically floating on air as they skate with < 5% of their leg area touching the water surface. This enables them to easily detach from the water surface during jumping. Another consequence of superhydrophobicity is that sea sprays and rain drops do not wet *Halobates*—the droplets simply bounce or roll off. Furthermore, in case of accidental submersion underwater, the microtrichia trap a thin film of air (known as a plastron), allowing the insects to breathe underwater for extended periods of time [21].

Oceanic *H*. *germanus* and coastal *Halobates hayanus* are superhydrophobic and up to 4 times smaller and 2 to 3 times lighter than their freshwater relatives, yet their leg lengths are similar (Fig 3) [29]. These adaptations allow *Halobates* to fully harness the surface tension of seawater; for instance, by pressing on the water surface, they can launch themselves at impressive vertical accelerations of approximately 400 ms^−2^, which is several times faster than the reported acceleration of their freshwater counterparts (viz *Aquarius paludum* at approximately 70 ms^−2^) [33]. The smaller body size of *Halobates* also results in a larger plastron volume relative to body weight, thereby increasing underwater resilience and buoyancy [29], enabling the insects to resurface quickly from accidental submersion. These are some of the crucial physiological adaptations that enabled *Halobates* to thrive under harsh marine conditions. Although we have uncovered some of their special adaptations, many mysteries remain to be solved (Box 1).

**Box 1. Unsolved mysteries surrounding *Halobates***

**Maneuvering rough waters**

Given that water does not stick to superhydrophobic legs of *Halobates*, how do they maneuver on the dynamic ocean surface without slipping or spinning? Even if they manage to anchor to the water surface, how do they disengage prior to jumping off? A related freshwater skater *Gerris remigis* has long thin hairs with pointed tips that are aligned to form V-shaped spacing between them that expels condensed water droplets from their body surface under humid conditions [34]. Whether *Halobates* has conserved this feature during its evolution from freshwater ancestors remains to be explored.

**Wax secretions**

Grooming performed by *Halobates* is expected to facilitate the spreading of waxy secretions produced by its meta-thoracic gland. Identification of the chemical constituents of these secretions and their specific contribution to coating the microhairs and nanohairs that cover *Halobates* to bestow water repellence as well as protection from UV and IR radiation remains to be explored.

**UV and IR protection**

With a small adult body, long thin legs, and no wings, *Halobates* can neither fly nor dive. Being confined to the ocean surface, they are exposed to breaking waves, torrential rainstorms, and hurricane winds at all times. Unlike desert beetles, which can dive under the sand to avoid heat from the noonday sun, how do *Halobates* avoid UV radiation damage or overheating with nowhere to seek shade or hide? It is known that oceanic *H*. *sericeus* has a highly UV-absorbing layer that prevents <0.0002% of UV at 280 nm to be transmitted through its cuticle, whereas its freshwater relative *Gerris* allows approximately 50% of UV to pass through its cuticle [35], the nature of this layer remains to be determined.

**How do they find each other?**

Living in a strictly 2D habitat with their body and eyes raised barely 1 mm above the ocean surface, what *Halobates* see on a calm day would be a completely flat surface extending over a seemingly limitless horizon. How to they find food and mates or avoid fish and other predators from the water below or seabirds from the sky above? Can they sense prey and predators by vibrations on the sea surface or see shadows cast by seabirds? Do they have surface-dispersible pheromones to attract mates, as has been shown in a related marine veliid [36]? When cast afar by winds and currents beyond their comfort zone to where surface seawater temperatures are above 20°C or below 30°C, are they able to navigate and return “home”? These mysteries all remain to be solved.

## Environmental concerns—Can *Halobates* help?

*Halobates* is the sole animal known to live at the ocean surface. With a distribution over half of the oceans that cover 70% of the Earth’s surface, they must be one of the most widely distributed organisms in the world. They can be caught easily with a neuston net and are extremely easy to distinguish from all other marine animals by their black body and long thin legs. It is quite conceivable that they can be used as indicators for environmental studies of global concerns.

### Plastics and heavy metals in the ocean

As ocean skaters are confined to the 2D sea surface, they encounter floating plastic debris and pollutants that accumulate at the air–water interface. A transect carried out between San Diego and Hawaii revealed that there was a 10-fold increase in plastic debris over a 40-year period [37]. Could the use of this increased volume of plastic debris as an egg-laying substrate enhance the reproduction and increase the population of *Halobates*? It is also interesting to note that heavy metals and other toxic chemicals are known to accumulate at the ocean surface and are taken up by *Halobates* [38,39]. Given that *Halobates* are ubiquitous on major ocean surfaces and easy to catch and identify, could it prove to be a useful indicator of plastic and heavy metal pollution?

### Climate change and ocean warming

The Red Sea is one of the hottest seas on Earth and can be used for real-life simulation studies of climate change and ocean warming. For example, a super coral with a specialized holobiome that is able to withstand heat and prevent bleaching has been found in the northern Red Sea [40]. How the Red Sea *Halobates*, which can tolerate summer seawater temperatures of >30°C, interact with and recruit their microbiome from their habitat may help us understand how marine life might cope with increasing global temperatures.

### Bio-inspirations

*Halobates* are able to robustly entrap air at the liquid–solid interface to perform effortless skating on water, ultrafast jumping from the water surface, and underwater respiration. These strategies, which combine surface texture and chemical makeup, could lead to rational designs of materials and technologies, such as water-repellent surfaces, pipes with low frictional drag, and robust liquid–vapor separation. Preliminary investigations of bio-mimicking microtextures with mushroom-shaped pillars [32,41] and cavities [42,43] have demonstrated that it is possible to achieve air entrapment underwater without chemical coatings. These gas-entrapping microtextured surfaces show promise for mitigating cavitation erosion in fluid machinery [44]. Along these lines, a proof-of-concept demonstration of coating-free gas-entrapping membranes for water desalination from warm saline feeds has also been reported [45,46]. Multidisciplinary research is needed for evaluating the pros and cons of these new bio-inspired approaches, including their effects on drag reduction, and the replenishment of entrapped air [47]. In-depth studies of protection from UV radiation could also inspire new approaches for UV-repellent surfaces and coatings.

## Conclusions

Insects are immensely adaptable and are the most successful animals on land. So why did only one genus of insects, *Halobates*, take to the high seas? Perhaps by the time *Halobates* evolved some 45 Mya, the only marine habitat remaining to be conquered was the open ocean, where the physical conditions were too challenging for other organisms to overcome. We can imagine one or more gravid females of an ancestral coastal *Halobates*, already predisposed to live on the sea surface, being carried by the wind out to the open sea. Unable to return to coastal waters to feed, they might initially have found terrestrial insects carried by the wind far from land and deposited at sea to feed on [48,49], then maybe later adapted to feed on zooplankton caught at the sea surface. In the absence of attached substrates for oviposition, they must have found floating material to lay eggs on. The young nymphs that hatched at sea somehow managed to survive, molt, became adults, found mates, multiply, and eventually took over the open oceans.

The evolutionary steps that were taken by ancestral *Halobates* to survive on the high seas remain unknown. We postulate that the prerequisites for ocean life are the complete loss of wings, enabling them to divert energy toward the development of strong leg muscles, and newly hatched nymphs being completely independent and able to move and feed. Based on what we have learned about *Halobates* so far, crucial adaptations for open ocean life include a reduction in body size and shape to better survive rainstorms and breaking waves; superhydrophobicity for preventing wetting and drowning; the development of a robust plastron enabling respiration underwater during accidental submergence; strong leg muscles coupled with superhydrophobicity, enabling them to realize impressive acceleration and agility for avoiding predators, raindrops, and breaking waves; and a highly reflective and protective body surface to avoid damaging effects from UV and IR radiation.

A greater fundamental understanding of these adaptations is clearly needed. Ongoing research into how coastal species compare with open ocean species will hopefully not only shed light on the unsolved mysteries surrounding *Halobates* but also provide us with ideas for the development of bio-inspired technologies. With modern advances in gene sequencing techniques, we also hope to eventually unlock secrets of their adaptive genes that may allow us to develop UV and IR protective materials. These are perhaps pipe dreams, but who knows what the future holds?
